# Gene therapy via CRISPR/Cas9-mediated *Cxcr4* disease allele inactivation reverses leukopenia in WHIM mice

**DOI:** 10.1172/JCI202073

**Published:** 2026-01-08

**Authors:** Ji-Liang Gao, Zhanzhuo Li, Rafael Calderon-Perez, Antonia Pavek, Lina Kim, David H. McDermott, Philip M. Murphy

**Affiliations:** Molecular Signaling Section, Laboratory of Molecular Immunology, National Institute of Allergy and Infectious Diseases (NIAID), NIH, Bethesda, Maryland, USA.

**Keywords:** Hematology, Immunology, Gene therapy

## Abstract

Warts, hypogammaglobulinemia, infections, and myelokathexis (WHIM) syndrome is an immunodeficiency caused by autosomal dominant hyperfunctional mutations in chemokine receptor *CXCR4* that promote panleukopenia due to BM retention. We previously reported a preclinical gene therapy protocol involving allele-nonspecific *Cxcr4* CRISPR/Cas9 inactivation, leveraging the known in vivo dominance of *Cxcr4^+/o^* (+, WT; o, inactivated) hematopoietic stem cells (HSCs) for autologous BM engraftment and leukocyte reconstitution over HSCs with other *Cxcr4* genotypes. Here, we show that without BM conditioning, this approach is not able to correct leukopenia in WHIM mice. We therefore modified the protocol by adding conditioning with a nongenotoxic CD117-targeted immunotoxin, CD117-antibody-saporin-conjugate. With this change, donor-derived blood cells rapidly reached ~95% chimerism after transplantation, which was stable without adverse events. Mice receiving edited HSCs showed rapid normalization of absolute myeloid cell counts, the key blood subset responsible for WHIM syndrome. In competitive transplants using equal numbers of edited and unedited donor HSCs, over 80% of blood cells originated from the edited population, predominantly with the *Cxcr4^+/o^* genotype. These results provide proof of principle that CRISPR/Cas9-mediated inactivation of the *Cxcr4* disease allele, combined with nongenotoxic HSC-targeted conditioning, may offer a safe and effective gene therapy strategy generalizable to all WHIM-causing mutations.

## Abstract 2

CRISPR silencing of an overactive disease allele corrected leukopenia in a murine model of WHIM syndrome, demonstrating a new therapeutic strategy for dominant immune disorders.

## Introduction

Warts, hypogammaglobulinemia, infections, and myelokathexis (WHIM) syndrome is a rare, autosomal dominant primary immunodeficiency disorder caused by mutations in the *CXCR4* gene (1, 2), which encodes a chemokine receptor critical for hematopoietic cell trafficking, proliferation, and retention in the BM (3–7). These mutations typically truncate the C-terminal tail of CXCR4, leading to impaired receptor desensitization and enhanced signaling in response to its ligand, CXCL12 (8, 9). As a result, mature leukocytes — particularly neutrophils — are abnormally retained in the BM, a phenomenon known as myelokathexis, leading to chronic neutropenia (and panleukopenia in most cases) and increased susceptibility to recurrent bacterial and viral infections, usually at barrier sites (10–13).

Current treatments for WHIM syndrome, including granulocyte colony-stimulating factor, immunoglobulin replacement, and the specific CXCR4 antagonists plerixafor and mavorixafor, require lifelong administration and may cause adverse effects in some patients (11, 14–17). While allogeneic hematopoietic stem cell (HSC) transplantation is curative, application is limited by donor availability and by risks from the procedure, including graft-versus-host disease (GVHD) and side effects from genotoxic BM conditioning, which are not justifiable for most cases of WHIM syndrome (18). Gene therapy using autologous transplantation of patient HSCs corrected ex vivo obviates the risk of GVHD but not genotoxic conditioning.

Our previous work developed a 2-step gene therapy strategy for WHIM model mice, which we designate as *Cxcr4^+/w^* (+, WT; w, WHIM). This involved ex vivo CRISPR/Cas9 inactivation of *Cxcr4* in heterozygous *Cxcr4^+/w^* HSCs using a guide RNA that does not distinguish between the WHIM allele and the WT allele, followed by preferential in vivo hematopoietic reconstitution of edited HSCs where only the WHIM allele had been inactivated, i.e., hemizygous *Cxcr4^+/o^* cells (19). The protocol takes advantage of the normal function of Cxcr4 to promote HSC quiescence and leukocyte retention in BM, such that less Cxcr4 signaling in *Cxcr4^+/o^* cells favors increased HSC proliferation and less leukocyte-retentive activity, thereby favoring release of mature leukocytes into the blood (5). Together, in the context of transplantation, these activity shifts provide a competitive advantage for reconstitution of *Cxcr4^+/o^* cells over leukocytes with other *Cxcr4* genotypes. We demonstrated this in multiple ways, first by competitive transplantation experiments using mixtures of HSCs from *Cxcr4^+/o^* and *Cxcr4^+/w^* mice as donor cells for both lethally irradiated and unconditioned *Cxcr4^+/w^* recipient mice (20, 21), then by experiments in *Cxcr4^+/o^*:*Cxcr4^+/w^* parabiotic mice (22), and finally by using mixtures of *Cxcr4*-edited and mock-edited HSCs from *Cxcr4^+/w^* donor mice transplanted into lethally irradiated *Cxcr4^+/w^* recipient mice (19). This strategy has the advantage of being universal for all *CXCR4* mutations. Regarding safety, *Cxcr4^+/o^* mice have a normal lifespan (21). Regarding the potential for safe clinical translation, we previously reported a long-lived patient with WHIM who appeared to be spontaneously cured of the disease by an analogous genetic event: en bloc chromothriptic deletion of the WHIM allele in a single HSC (20).

In the present study, we extend these findings by developing a clinically feasible gene therapy strategy for WHIM syndrome based on CRISPR/Cas9-mediated inactivation of the disease allele in autologous HSCs. When combined with nongenotoxic conditioning, this approach enabled durable engraftment, robust multilineage reconstitution of edited HSCs, and effective correction of leukopenia in WHIM mice. These results advance the translational potential of gene therapy for WHIM syndrome and provide a roadmap toward a curative autologous HSC-based treatment.

## Results

### Enhanced but limited engraftment of Cxcr4-edited HSCs in unconditioned WHIM mice.

We previously demonstrated that leukopenia in WHIM mice can be corrected by transplanting *Cxcr4^+/o^* BM cells without recipient conditioning, but correction required transplantation of 50 million total BM cells to achieve 70% myeloid chimerism in the blood (21). More recently, we developed a CRISPR/Cas9-based protocol to inactivate the disease allele in donor HSCs and demonstrated a selective advantage of *Cxcr4^+/o^* cells over *Cxcr4^+/w^* cells for hematopoietic reconstitution (19). We sought to determine whether this gene-editing approach could generate a sufficient number of *Cxcr4^+/o^* cells to correct leukopenia in WHIM mice without conditioning.

**Figure 1:**
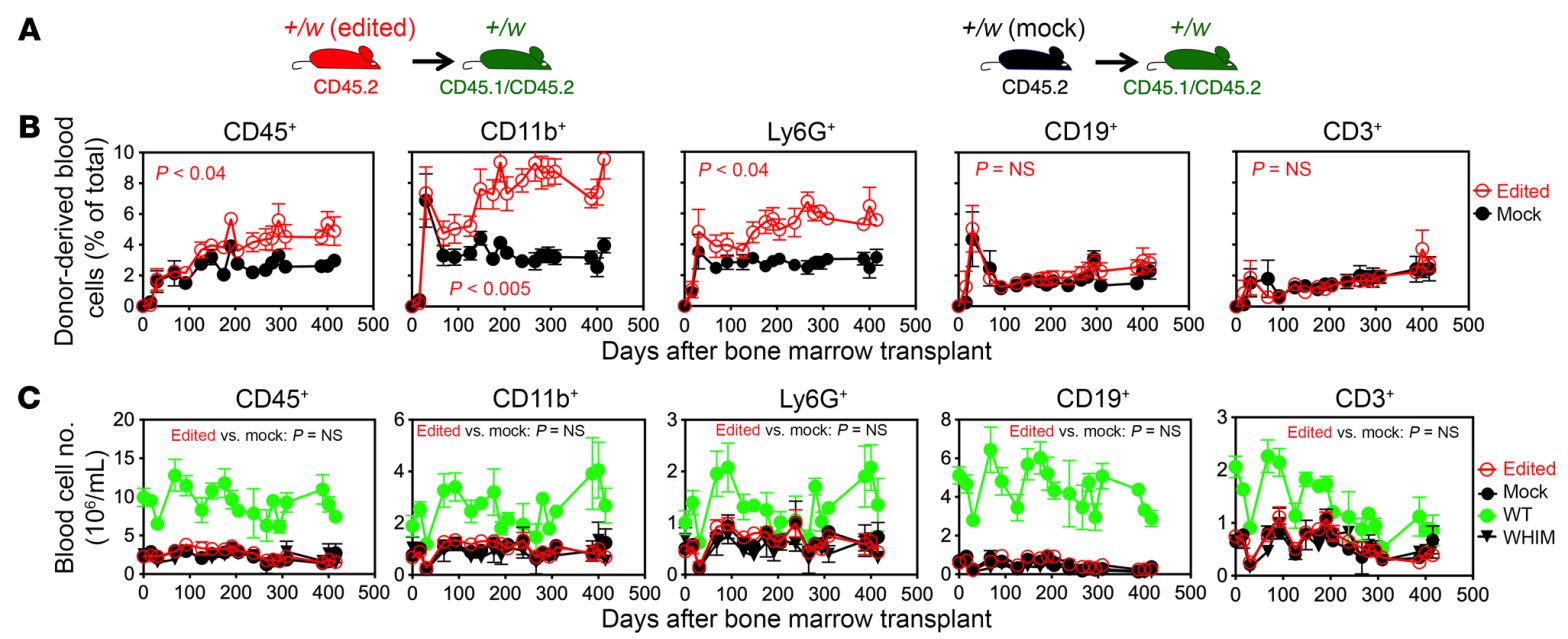
Enhanced but limited engraftment of *Cxcr4*-edited HSCs in unconditioned WHIM mice. (**A**) Experimental design. Unconditioned *Cxcr4^+/w^* (+/w) mice were transplanted with 10^6^ *Cxcr4*-sgRNA/Cas9 RNP-transfected [+/w (edited)] or mock-transfected [+/w (mock)] *Cxcr4^+/w^* HSPCs (cKit^+^) (*n* = 5 per group). Donor and recipient mice were marked genetically by the indicated CD45 polymorphisms. (**B** and **C**) Time course of donor-derived leukocyte reconstitution in recipient blood after transplantation as a percentage of total cells (**B**) and as absolute total cell counts (**C**) for each of the subsets indicated at the top of each column of panels. The symbol keys are to the far right. Untransplanted WT and untransplanted *Cxcr4^+/w^* (WHIM) control littermates were tested at the same time points. Two-way ANOVA was used for statistical comparison between 2 groups.

To test this, we transplanted 10^6^ *Cxcr4*-sgRNA/Cas9 ribonucleoprotein-electroporated (RNP-electroporated) cKit^+^ BM cells (HSPCs, hematopoietic stem and progenitor cells) from *Cxcr4^+/w^* mice (abbreviated *Cxcr4*-RNP-transfected HSPCs) into each *Cxcr4^+/w^* congenic recipient mouse and monitored donor cell engraftment in peripheral blood, along with total blood counts, over 400 days. For comparison, we set up 3 control groups: mock control (*Cxcr4^+/w^* mice transplanted with the same number of *Cxcr4^+/w^* HSPCs that were electroporated with Cas9 protein alone without sgRNA), untransplanted littermates of *Cxcr4^+/w^* mice, and untransplanted littermates of WT mice. To track transplanted donor cell fate in vivo, donor mice and recipient mice had distinct CD45 congenic markers, CD45.2 and CD45.1/CD45.2, respectively (Figure 1A).

In the mock control group, donor-derived cells accounted for only ~2% of each leukocyte subset in the peripheral blood of unconditioned *Cxcr4^+/w^* recipients. In contrast, recipients of *Cxcr4*-RNP-transfected donor cells showed significantly higher frequencies of donor-derived myeloid cells (CD11b^+^) and neutrophils (Ly6G^+^), reaching up to ~10% and ~8%, respectively (Figure 1B). However, this level of chimerism failed to significantly increase peripheral white blood cell counts compared with mock controls and untransplanted *Cxcr4^+/w^* littermates (Figure 1C).

### Nongenotoxic CD117-antibody-saporin-conjugate is a safe and effective conditioning reagent for durable hematopoietic reconstitution of donor Cxcr4^+/o^ HSCs to reverse leukopenia in WHIM mice.

We next evaluated whether our CRISPR/Cas9 editing protocol could generate sufficient numbers of *Cxcr4^+/o^* HSCs to correct leukopenia under clinically feasible conditions.

As an estimate, the expected genotype frequencies resulting from a gene-editing event can be calculated from the edited allele frequencies using the Hardy-Weinberg equation (p² + 2pq + q² = 1). This calculation assumes a population in Hardy-Weinberg equilibrium, where p represents the frequency of the edited allele and q the frequency of the unedited allele (i.e., q = 1 – p). Accordingly, the frequency of cells with both alleles edited (o/o) is p², the frequency of cells with both alleles unedited (+/+) is (1 – p)², and the frequency of heterozygous cells is 2p(1 – p) (Supplemental Figure 1; supplemental material available online with this article; https://doi.org/10.1172/JCI202073DS1) (23).

In the case of *Cxcr4^+/w^* cells, editing may occur on either the WT or WHIM allele (Supplemental Figure 1). Assuming that the editing probability is the same for both alleles, the frequency of *Cxcr4^o/o^* cells remains p², and the frequency of unedited cells (*Cxcr4^+/w^*) is (1 – p)². The heterozygous population is composed of 2 genotypes: *Cxcr4^o/w^* (WT allele edited) and *Cxcr4^+/o^* (WHIM allele edited). Since both are assumed to occur with equal probability, each accounts for half of the heterozygous frequency, i.e., 2p(1 – p)/2. Thus, the maximum achievable frequency of the *Cxcr4^+/o^* genotype that we can generate is 25% under our CRISPR/Cas9 editing protocol (Supplemental Figure 1).

Based on this estimate, to generate 50 million *Cxcr4^+/o^* donor BM cells — sufficient to correct leukopenia in an unconditioned *Cxcr4^+/w^* recipient as reported previously (21) — approximately 200 million *Cxcr4^+/w^* donor BM cells would be required at the outset of editing, a quantity that is clinically impractical.

This analysis highlights the need for additional strategies to improve the in vivo performance of gene-edited cells. To enhance engraftment, we evaluated gene therapy in combination with a nongenotoxic CD117-targeted conditioning reagent, CD117-antibody-saporin-conjugate (CD117-ASC). Saporin is a plant-derived ribosome-inactivating protein that halts protein synthesis, leading to cell death. A single dose of CD117-ASC has been shown to deplete more than 99% of host HSCs, enabling rapid and efficient donor hematopoietic cell engraftment in both autologous transplantation and allotransplantation protocols in WT mice (24, 25). Importantly, the absence of genotoxicity makes this approach particularly well suited for a relatively benign genetic disease like WHIM syndrome.

**Figure 2:**
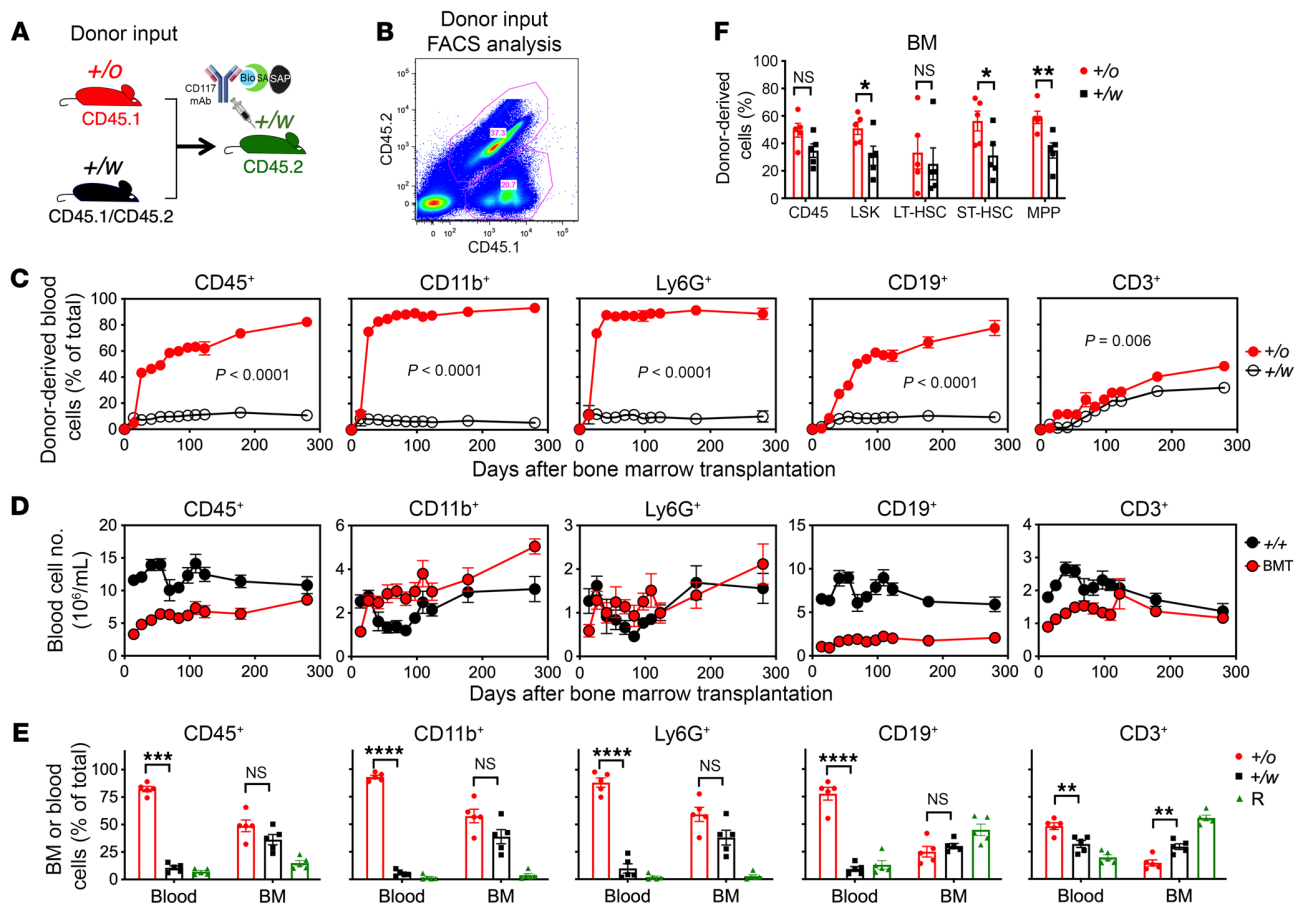
Nongenotoxic CD117-ASC is a safe and effective conditioning reagent for durable hematopoietic reconstitution of donor *Cxcr4^+/o^* HSCs to reverse leukopenia in WHIM mice. (**A**) Experimental design of competitive transplantation. Recipient *Cxcr4^+/w^* (+/w) mice were injected i.v. with CD117-ASC 8 days before transplantation and then transplanted i.v. with a 1:1 mixture of 5 × 10^6^ BM cells each from *Cxcr4^+/o^* (+/o) and *Cxcr4^+/w^* (+/w) donor mice. Donor and recipient mice were marked genetically by the indicated CD45 polymorphisms. Bio, biotin; SA, streptavidin; SAP, saporin. (**B**) Flow cytometry plot of input mixed donor cells. (**C** and **D**) Time course of donor-derived leukocyte reconstitution in recipient blood after transplantation as a percentage of total cells (**C**) and as absolute total cell counts (**D**) for each subset indicated at the top of each column of panels. The symbol keys are to the far right of each row of panels. BMT, BM transplanted; +/+, WT littermates. (**E**) Mature donor-derived BM cells 280 days after transplantation. The corresponding blood frequencies are replotted from panel **C** for each subset to facilitate comparison. R, recipient. (**F**) Donor-derived CD45^+^ cells and HSPCs in BM 280 days after transplantation. LSK, Lin^–^Sca1^+^cKit^+^; LT-HSC, long-term hematopoietic stem cell (CD34^–^Flt3^−^Lin^−^Sca1^+^cKit^+^); ST-HSC, short-term hematopoietic stem cell (CD34^+^Flt3^−^Lin^−^Sca1^+^cKit^+^); MPP, multipotential progenitor (CD34^+^Flt3^+^Lin^−^Sca1^+^cKit^+^). Data are from a single experiment (*n* = 5 mice per data point), representative of 2 independent experiments. In **E** and **F**, 2-tailed Student’s *t* test was used for statistical comparison between 2 groups. *P < 0.05; **P < 0.01; ***P < 0.005; ****P < 0.0001.

We first conducted competitive transplantation experiments using 5 × 10^6^ BM cells each from *Cxcr4^+/o^* and *Cxcr4^+/w^* mice as donor cells, with CD117-ASC–conditioned *Cxcr4^+/w^* mice as recipients (Figure 2). The transplanted mice showed no overt clinical abnormalities, such as increased mortality, body weight loss, spontaneous infections, or tumors. For all leukocyte subsets analyzed, *Cxcr4^+/o^* chimerism in peripheral blood was significantly higher than *Cxcr4^+/w^* chimerism. Notably, *Cxcr4^+/o^* myeloid cells reconstituted most efficiently, consisting of up to 95% of total myeloid cells in recipient mice (Figure 2C). The *Cxcr4^+/o^* advantage was maintained up to 281 days posttransplantation when the experiment was terminated. When mice were sacrificed, we observed a significant increase in the frequency of *Cxcr4^+/o^* donor-derived HSPCs (Figure 2F) but not mature leukocytes in the BM compared with the input frequency (Figure 2E). Most importantly, *Cxcr4^+/o^* BM transplantation corrected leukopenia in recipient *Cxcr4^+/w^* mice that had received BM CD117-ASC conditioning (Figure 2D). Thus, CD117-ASC appeared to be a safe and effective conditioning reagent for selective engraftment of *Cxcr4^+/o^* HSCs, consistent with its performance in autologous and allogeneic WT mouse HSC transplantation.

### CRISPR/Cas9-mediated WHIM allele inactivation corrects myeloid cytopenia in WHIM mice conditioned with CD117-ASC.

**Figure 3:**
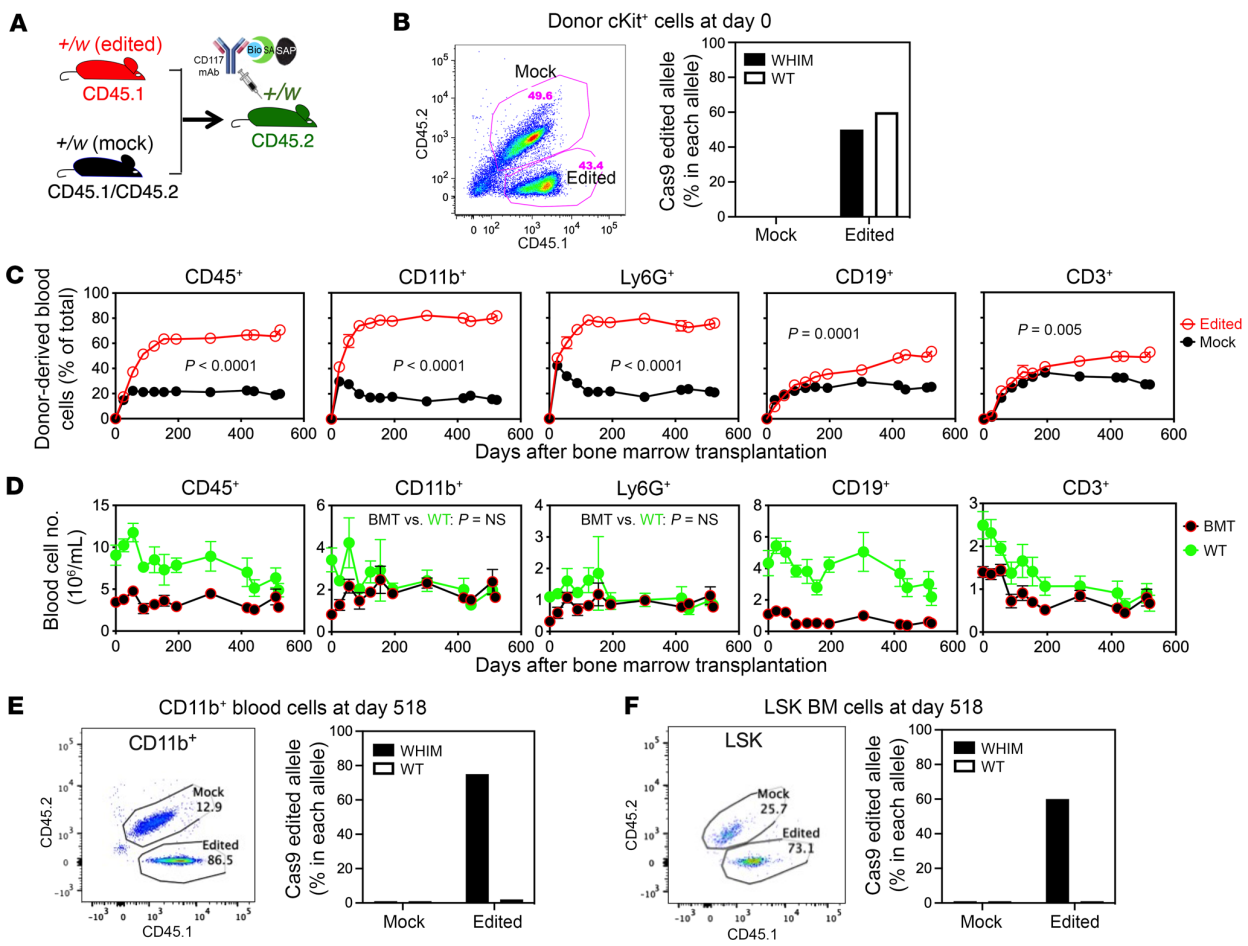
Nongenotoxic CD117-ASC is a safe and effective conditioning reagent for selective engraftment of *Cxcr4^+/o^* HSCs after allele-nonselective *Cxcr4* inactivation by CRISPR/Cas9. (**A**) Experimental design of competitive transplantation. Recipient *Cxcr4^+/w^* (+/w) mice were injected i.v. with CD117-ASC 8 days before transplantation and then transplanted i.v. with a mixture of 5 × 10^5^ each of *Cxcr4*-sgRNA/Cas9 RNP-transfected [+/w (edited)] and mock-transfected [+/w (mock)] HSPCs (cKit^+^) from *Cxcr4^+/w^* donor mice (*n* = 5 per recipient group). Donor and recipient mice were marked genetically by the indicated CD45 polymorphisms. Bio, biotin; SA, streptavidin; SAP, saporin. (**B**) Flow cytometry plot of input mixed donor cells (left) and frequencies of Cas9-edited (*Cxcr4* inactivated) alleles identified by PCR sequencing of the donor cells (right). (**C** and **D**) Time course of donor-derived leukocyte reconstitution in recipient blood after transplantation, shown as percentages of total cells (**C**) and as absolute total cell counts (**D**) for each subset indicated at the top of each column of panels. The symbol keys are shown at the far right. Untransplanted WT controls and transplanted littermates (BMT) were tested at the same time points. (**E** and **F**) *Cxcr4* editing analysis of flow cytometry–sorted CD11b^+^ cells from blood (**E**) and LSK cells from BM (**F**) at 518 days posttransplantation. Left panels: representative flow cytometry plots of sorted populations from a recipient mouse. Right panels: frequencies of Cas9-edited (*Cxcr4*-inactivated) alleles identified by PCR sequencing of the sorted cells. In **D**, 2-way ANOVA was used for statistical comparison between 2 groups.

We next tested engraftment of *Cxcr4*-RNP-transfected *Cxcr4^+/w^* HSPCs in CD117-ASC-conditioned WHIM mice. To do this, we first conducted competitive transplantation using a 1:1 mix of 5 × 10^5^ each of *Cxcr4*-RNP-transfected and mock-transfected *Cxcr4^+/w^* HSPCs as competitive donor cells (Figure 3A).

The editing efficiency of the donor cells was assessed using a T7E1 assay (data not shown) and DNA sequencing of cloned *Cxcr4* PCR amplicons that covered both the protospacer adjacent motif site targeted by CRISPR/Cas9 and the WHIM mutation site, allowing unambiguous assignment of indels to either the WT or WHIM allele. Among 9 PCR clones derived from the DNA of *Cxcr4*-RNP-transfected input HSPCs, 3 of 5 WT alleles and 2 of 4 WHIM alleles were edited. The overall allele-editing efficiency was approximately 50%, as we have observed previously using the same protocol. As expected, none of the 10 PCR clones from the DNA of mock-transfected input HSPCs showed evidence of CRISPR/Cas9-mediated editing (Figure 3B).

Following transplantation, we monitored hematopoietic reconstitution over time by FACS analysis of peripheral blood. Transplanted mice exhibited no overt clinical abnormalities. Results revealed strong donor cell engraftment and a strong selective advantage of *Cxcr4*-RNP-transfected donor cells over mock-transfected cells in reconstituting blood leukocytes. Frequencies of *Cxcr4*-RNP-transfected donor-derived leukocytes were significantly higher across all tested subsets compared with their initial input frequencies. Notably, myeloid cells derived from *Cxcr4*-RNP-transfected donors constituted up to 87% of total myeloid cells in peripheral blood (Figure 3, C and E). This advantage was durable and stable until the end of the experiment (day 518 posttransplantation).

When the mice were sacrificed, the frequency of *Cxcr4*-RNP-transfected donor-derived LSK (lineage^–^Sca1^+^cKit^+^) cells was also significantly increased in the BM relative to the input cell frequency, albeit to a lesser extent than the frequency of donor-derived cells in peripheral blood (Figure 3F).

Since the sgRNA we used targets a nonmutated region of *Cxcr4* and is therefore not selective for the WHIM allele over the WT allele, we next quantitated in vivo enrichment of WHIM allele-inactivated donor-derived *Cxcr4^+/o^* cells after transplantation. The cells could be derived from either *Cxcr4*-RNP-transfected or mock-transfected donors (Figure 3E). DNA from CD11b^+^ cells sorted from the blood at day 518 posttransplantation was analyzed using the T7E1 assay and sequencing of cloned *Cxcr4* PCR amplicons. Among 9 PCR clones from Cxcr4-RNP-transfected donor cells (from 1 mouse), 3 of 4 WHIM alleles, but none of 5 WT alleles, were edited by CRISPR/Cas9. As expected, none of 9 clones derived from the mock-transfected donor cells showed indels (Figure 3E). These results were confirmed by T7E1 assays from 5 individual mice (data not shown). Since no WT allele was edited, and further assuming that no cells had the *Cxcr4^o/o^* genotype (*Cxcr4^o/o^* HSCs are unable to engraft in BM after transplantation; ref. 5), the results indicated that approximately 75% of CD11b^+^ cells derived from *Cxcr4*-RNP-transfected donors carried inactivated WHIM alleles. Given that these donor-derived CD11b^+^ cells made up 87% of the recipient CD11b^+^ cells (Figure 3E), the final proportion of WHIM allele-inactivated CD11b^+^ cells was approximately 65% (75% of 87%).

We next assessed the frequency of WHIM and WT allele-inactivated cells in BM by analyzing FACS-purified LSK cells derived from *Cxcr4*-RNP-transfected and mock-transfected donor HSCs (Figure 3F). *Cxcr4* PCR amplicons from sorted cells were sequenced to identify indel frequencies in the WT or WHIM allele. Among 10 PCR clones from *Cxcr4*-RNP-transfected cells, 3 of 5 WHIM alleles, but none of the 5 WT alleles, were edited. As expected, none of 9 clones from mock-transfected cells contained indels. Since *Cxcr4*-RNP-transfected donor-derived LSK cells accounted for 73% of the total LSK cells in BM (Figure 3F), the final proportion of WHIM allele-inactivated LSK cells was approximately 44% (60% of 73%).

Importantly, the numbers of total CD11b^+^ myeloid cells and neutrophils in the peripheral blood of the transplanted *Cxcr4^+/w^* mice were sustained at a level similar to the range observed for control untransplanted WT littermates of the same age (Figure 3D).

**Figure 4:**
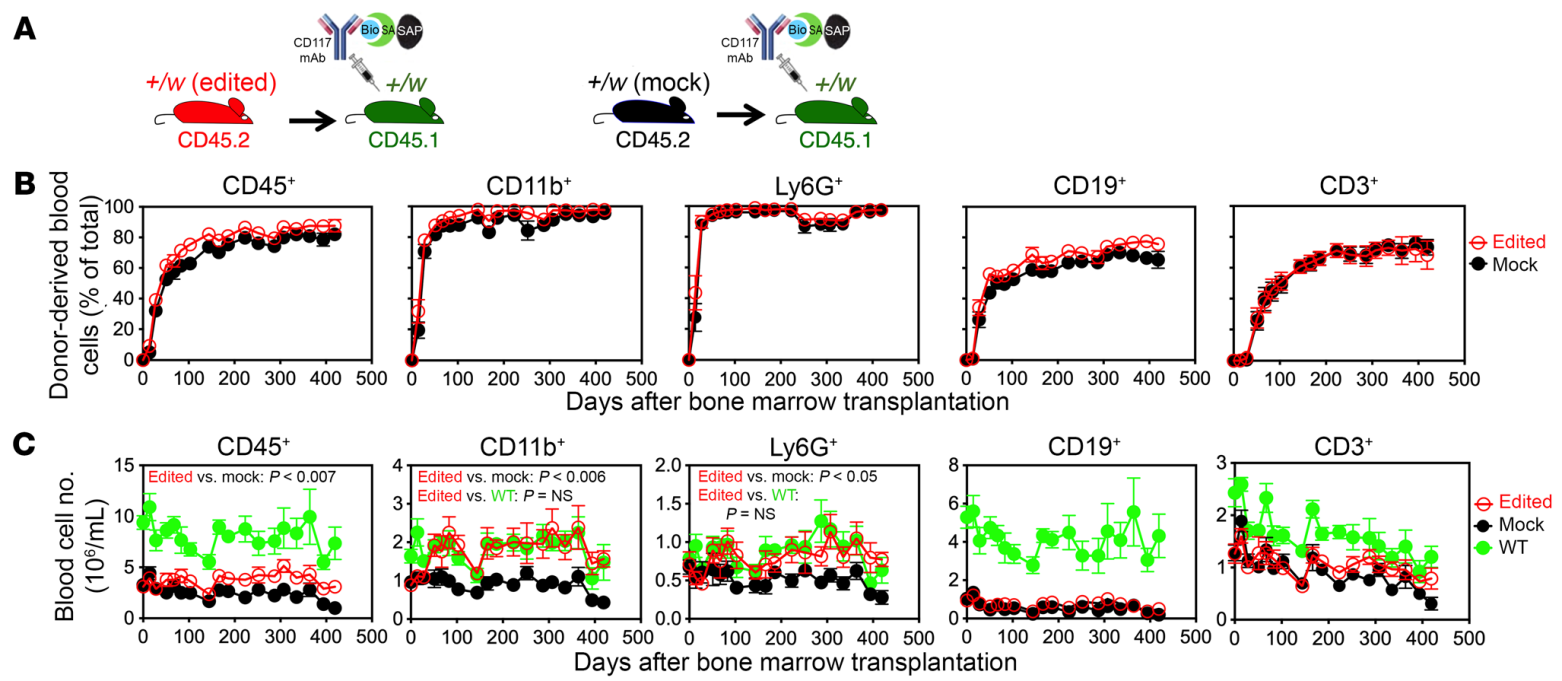
CRISPR/Cas9-mediated HSC WHIM allele inactivation corrects myeloid cytopenia in WHIM mice conditioned with CD117-ASC. (**A**) Experimental design. Recipient *Cxcr4^+/w^* (+/w) mice were injected i.v. with CD117-ASC 8 days before transplantation and then transplanted with 1.25 × 10^6^ Cxcr4-sgRNA/Cas9 RNP-transfected [+/w (edited)] or mock-transfected [+/w (mock)] *Cxcr4^+/w^* HSPCs (cKit^+^) (*n* = 6 per group). Donor and recipient mice were marked genetically by the indicated CD45 polymorphisms. Bio, biotin; SA, streptavidin; SAP, saporin. (**B** and **C**) Time course of donor-derived leukocyte reconstitution in recipient blood after transplantation as a percentage of total cells (**B**) and as absolute total cell counts (**C**) for each of the subsets indicated at the top of each column of panels. The symbol keys are to the far right. Unconditioned and untransplanted WT controls and transplanted littermates were tested at the same time points. Data are from a single experiment representative of 2 independent experiments. In **C**, 2-way ANOVA was used for statistical comparison between 2 groups.

We next tested single donor engraftment in CD117-ASC–conditioned WHIM mice (Figure 4). A total of 1.25 × 10^6^ *Cxcr4*-RNP-transfected or mock-transfected HSPCs from *Cxcr4^+/w^* mice were transplanted into each *Cxcr4^+/w^* recipient and monitored for donor cell engraftment in peripheral blood, along with total blood counts, for 418 days, when the experiment was terminated. Transplantation of either *Cxcr4*-RNP-transfected or mock-transfected cells into conditioned *Cxcr4^+/w^* mice quickly established donor cell engraftment in peripheral blood with ~90% for myeloid cells and ~75% for both B and T lymphocytes that was sustained at these levels for the entire time course of the experiment (Figure 4B). Importantly, the numbers of total myeloid cells and neutrophils in the peripheral blood of conditioned *Cxcr4^+/w^* mice receiving *Cxcr4*-RNP-transfected HSPCs were in the range of their untransplanted WT littermates of the same age, indicating full correction (Figure 4C). In contrast, blood leukocyte counts in conditioned *Cxcr4^+/w^* mice receiving mock-transfected HSCs were in the range of untransplanted *Cxcr4^+/w^* littermates of the same age.

## Discussion

In this study, we demonstrate that when combined with nongenotoxic conditioning, our protocol for CRISPR/Cas9-mediated inactivation of the WHIM allele in autologous HSPCs can generate a therapeutically meaningful proportion of circulating *Cxcr4^+/o^* cells in vivo. Although edited cells showed a modest engraftment advantage (~10%) in unconditioned WHIM mice, this was insufficient to reverse leukopenia. In contrast, when recipient mice were conditioned with CD117-ASC, edited donor cells exhibited robust, multilineage reconstitution, durable engraftment, and selective expansion. Most importantly, leukocyte counts — especially neutrophils and monocytes — were restored to WT levels, indicating functional correction of WHIM-associated leukopenia.

These findings build directly on our prior work, which established the foundational rationale for this gene therapy strategy targeting *CXCR4*. In a clinical observation, we reported that a patient with WHIM (WHIM-09) was spontaneously cured following chromothriptic deletion of the disease allele in a single HSC, which subsequently repopulated the myeloid lineage. This naturally occurring somatic reversion suggested that selective loss of the disease allele confers a strong in vivo competitive advantage and can resolve the primary clinical manifestations of the syndrome (20). We later confirmed this concept experimentally by showing that *Cxcr4^+/o^* donor HSCs outcompeted WHIM cells and normalized leukocyte counts in WHIM mice (21, 22). Building on these insights, we developed a CRISPR/Cas9-based strategy to recapitulate this therapeutic genotype through targeted inactivation of the WHIM allele in donor HSPCs (19).

A key strength of this strategy is its universality. Unlike mutation-specific correction approaches, which require customized protocols for each mutation, CRISPR/Cas9-mediated inactivation of *CXCR4* is applicable across all known WHIM-causing mutations — currently at least 35 distinct variants (26) — providing a broadly translatable therapeutic platform.

While edited *Cxcr4^+/o^* HSCs consistently outcompete WHIM cells, our data reveal a critical limitation: The absolute number of edited cells required for hematologic correction exceeds what is feasible with current CRISPR/Cas9 editing efficiencies. Unlike WHIM-09, in whom a single reverted HSC repopulated the myeloid lineage, edited cells in our model could not fully normalize hematopoiesis without conditioning. Since the chromothriptic event in WHIM-09 deleted 163 additional linked genes, the precise repopulation mechanism is not fully understood, and the unusually high efficiency of correction may have been influenced by additional genetic changes (20). Based on our estimate of the maximum achievable editing rate (~25%), achieving 5 million edited cKit^+^ cells (sufficient to match therapeutic levels used in prior studies; ref. 21) would require starting with approximately 200 million donor BM cells — an impractical target for clinical translation. These results highlight the need for additional strategies to augment the in vivo performance of gene-edited cells.

To address this, we employed a mild conditioning approach using CD117-ASC to transiently deplete endogenous HSPCs and create niche space in the BM for the engraftment and expansion of edited cells (24, 25). This strategy proved highly effective. CD117-ASC conditioning enabled efficient and durable engraftment of *Cxcr4^+/o^* HSCs, leading to long-term multilineage hematopoietic reconstitution. The edited cells not only engrafted more effectively but also expanded preferentially over time, consistent with their inherent selective advantage. Notably, frequencies derived from WHIM allele–edited donor cells were significantly increased among both blood leukocytes and BM LSK cells, further supporting successful niche colonization (Figure 3).

Compared with traditional conditioning regimens, such as total body irradiation or chemotherapy, CD117-ASC offers a targeted, nongenotoxic alternative that minimizes off-target tissue damage. CD117-ASC selectively binds to the cKit receptor on host HSPCs, delivering saporin to induce apoptosis without harming nonhematopoietic tissues (24, 25). This selectivity reduces the risk of infertility, organ toxicity, and secondary malignancies associated with genotoxic conditioning.

However, CD117-ASC conditioning did not allow donor *Cxcr4^+/o^* HSCs to fully correct lymphopenia in WHIM recipients (Figure 3D and Figure 4C), in contrast with results with lethal irradiation (22). This discrepancy may reflect competition from host lymphocytes (Figure 2E), which are not depleted by CD117-ASC and may limit production of donor-derived *Cxcr4^+/o^* lymphocytes. This possibility will be explored in future studies, though myeloid cells remain the key pathogenic subset in WHIM syndrome (20).

There are several limitations to our study. The long-term safety of CRISPR/Cas9 editing in human HSPCs remains to be fully assessed, particularly with regard to off-target activity and potential toxicity. Similarly, while CD117-ASC has shown promise in preclinical studies and early-phase trials, it is not yet broadly validated in human clinical settings. Future work should evaluate this combined strategy in large animal models or humanized mouse systems to better predict translational outcomes, particularly with regard to WHIM syndrome, which is not immediately life-threatening for most patients. Further, our study did not evaluate functional immunity directly after gene therapy. WHIM syndrome appears to be primarily a defect of leukocyte trafficking rather than intrinsic effector function (2), and *Cxcr4^+/o^* mice have normal lifespan and overall health (21), which provide positive safety signals. However, additional work will be needed to assess the mice under stressed conditions, such as infectious challenges, and to assess effector functions of specific leukocyte subpopulations.

In conclusion, we establish a proof of principle for a curative autologous gene therapy approach in WHIM syndrome using CRISPR/Cas9-mediated disease allele inactivation. Importantly, we show that coupling gene editing with nongenotoxic CD117-ASC conditioning enables efficient engraftment and durable reconstitution of hematopoiesis with edited *Cxcr4^+/o^* cells, resulting in long-term correction of leukopenia. These findings provide a strong preclinical foundation for advancing this strategy toward clinical translation in patients with WHIM syndrome.

## Methods

### Sex as a biological variable.

Our study examined male and female animals, and similar findings are reported for both sexes. No sex-dependent differences were observed, consistent with our previous report (21).

### Mice.

WHIM (*Cxcr4^+/w^*) mice on a homozygous CD45.1 or CD45.2 background or a heterozygous CD45.1/CD45.2 background have been previously reported (20, 21). The mice were kept in a specific pathogen–free facility at NIH and were 6–8 weeks old at the time of transplantation.

### Mouse HSPC isolation.

Murine BM cells were isolated by flushing tibias and femurs with PBS (KD Medical). HSPCs (cKit^+^ or CD117^+^) were isolated using a CD117 MicroBead Kit and an autoMACS Separator from Miltenyi Biotec following the manufacturer’s instructions.

### Gene editing.

The gene-editing procedure was described previously (19). Freshly isolated HSPCs were cultured in X-Vivo 15 media (Lonza) supplemented with 2% FBS, 50 ng/mL SCF, 50 ng/mL TPO, 10 ng/mL IL-3, and 10 ng/mL IL-6. Cytokines were from PeproTech. After 2-hour culture at 37°C with 5% CO_2_, cells were collected, washed once with PBS, and resuspended into Buffer T from the Neon Transfection Kit (Invitrogen) at 5 × 10^5^ cells per 8 μL. The *Cxcr4*-sgRNA/Cas9 RNP complexes were prepared by mixing equal volumes of sgRNA (0.5 μg/μL, custom-made from Invitrogen) and Cas9 protein (1 μg/μL, PNA Bio) for 10–15 minutes at room temperature. Cells and RNP complexes were then mixed with a ratio of 8 μL cells and 2 μL RNP complexes and electroporated with a Neon Transfection System (1,700 V, 20 ms, 1 pulse). Electroporated cells were resuspended in X-Vivo 15 media supplemented with 1× Pen/Strep (Invitrogen) and used immediately for transplantation by mouse tail vein injection. For DNA isolation, electroporated cells were cultured 1 day in X-Vivo 15 with 2% FBS and the aforementioned cytokines. For mock-transfected controls, the conditions were the same except for the absence of sgRNA.

### Genomic DNA isolation.

Genomic DNA from HSPCs or flow cytometer–sorted cells was isolated using the DNeasy Blood & Tissue Kit (QIAGEN).

### PCR cloning and sequencing.

The PCR cloning and sequencing were described previously (19). A target sequence of 1,086 bp that covers both the WHIM mutation site and the CRISPR/Cas9 targeting site was amplified using the primers Fw2300 (5′-CTTTGCAGATATACACTTCTGATAAC-3′) and Rev3386 (5′-ATATGTCTTTGCATAAGTGTTAGCTG-3′). The PCR product was isolated using a PCR purification kit (QIAGEN), cloned with a PCR Cloning Kit (New England Biolabs), and sequenced by Eurofins (Eurofins Scientific). Sequence analysis was performed with Geneious Prime.

### T7E1 assay.

A *Cxcr4* amplicon of 423 bp that includes *Cxcr4*-sgRNA/Cas9 targeting site was generated using the primers F2382 (5′-GTGACGTTGTCTGTCCCTGT-3′) and R2700 (5′-AGGTACCGGTCCAGGCTGAT-3′). The PCR products were diluted 1:4 in 1× NEBuffer 2 (New England Biolabs) and hybridized slowly in a thermal cycler. Twenty microliters of hybridized fragments were then digested with 1.25 U of T7E1 (New England Biolabs) for 15 minutes at 37°C. Digested fragments were separated by agarose gel electrophoresis.

### Transplantation experiments.

For competitive donor transplantation, a mix of *Cxcr4*-sgRNA/Cas9 RNP-transfected and mock-transfected HSPCs, 5 × 10^5^ each, in 0.5 mL X-Vivo 15 media was injected by tail vein into sex-matched recipient WHIM mice. For single donor transplantation, ~10^6^ *Cxcr4*-sgRNA/Cas9 RNP-transfected or mock-transfected HSPCs in 0.5 mL X-Vivo 15 media were injected by tail vein into sex-matched recipient WHIM mice. For nongenotoxic conditioning, recipient WHIM mice were injected i.v. with 1 dose of CD117-ASC (1.2 mg/kg) 8 days before transplantation.

### Flow cytometry analysis.

For leukocyte subset analysis, 100 μL of mouse blood was collected and incubated for 10 minutes with 2 μL of Fc block (BioLegend) before incubation with the following monoclonal antibodies at 4°C for 30 minutes: CD45.1-PECy7 (clone A20) and CD45.2-eFluor450 (clone 104) (eBioscience), Ly6G-APC-Cy7 (clone lA8), CD11b-PerCP-Cy5.5 (clone M1/70), CD19-FITC (clone 6D5), and CD3-APC (clone 145-2C11). Erythrocytes were lysed with 3 mL ACK lysis buffer (Quality Biologicals) for 3 minutes at room temperature. Cells were then washed once with FACS buffer and analyzed using a Fortessa FACS cytometer (BD Biosciences) and FlowJo software (TreeStar). A similar procedure was followed for staining BM cells.

### Cell isolation for Cxcr4 genomic analysis.

CD11b^+^ cells derived from donor HSCs were isolated from blood of transplanted mice by positively sorting (FACSAria II, BD Biosciences) with antibodies directed against CD45.1-PECy7 (clone A20), CD45.2-eFluor450 (Clone 104) (eBioscience), and CD11b-PerCP-Cy5.5 (clone M1/70) (Biolegend). LSK cells derived from donor HSCs were isolated from BM of transplanted mice by lineage depletion with the Lineage Depletion Kit (Miltenyi Biotec), then by FACS sorting (FACSAria II) with antibodies directed against Sca1-APC-Cy7 (D7), cKit-APC (2B8), streptavidin-PE (catalog 405203) (Biolegend) (to target residual lineage positive cells after lineage depletion), CD45.1-PECy7 (clone A20) and CD45.2-eFluor450 (Clone 104) (eBioscience).

### Blood cell counts.

Blood was collected from mandibular veins of recipient mice using EDTA as an anticoagulant (Becton Dickinson). Total leukocyte counts were measured with a Cellometer Auto 2000 Cell Viability Counter (Nexcelom Bioscience). Absolute leukocyte subset counts and frequencies were quantitated by flow cytometry.

### Statistics.

Each data point is presented as the mean ± SEM. Two-tailed Student’s *t* test for single comparisons and 2-way ANOVA for multiple comparisons were used. *P* values less than 0.05 were considered significant.

### Study approval.

All animal experiments were performed using an NIAID Animal Care and Use Committee–approved protocol.

### Data availability.

Values for all data points in graphs are reported in the Supporting Data Values file.

## Author contributions

JLG, DHM, and PMM conceived and designed the study. JLG, ZL, RCP, AP, and LK performed the experiments. JLG and PMM analyzed the data and wrote the first draft of the manuscript, which was supplemented by all authors.

## Funding support

This work is the result of NIH funding, in whole or in part, and is subject to the NIH Public Access Policy. Through acceptance of this federal funding, the NIH has been given a right to make the work publicly available in PubMed Central.

- Intramural Research Program of the NIAID, NIH.

## Supplementary Material

Supplemental data

Supporting data values

## Version 1 01/08/2026

In-Press Preview

## Version 2 03/02/2026

Electronic publication
