# Supplementary material for: Gene therapy via CRISPR/Cas9-mediated Cxcr4 disease allele inactivation reverses leukopenia in WHIM mice
Source: J Clin Invest. 2026 Jan 8;136(5):e202073. doi: 10.1172/JCI202073 (PMC12948438; doi:10.1172/JCI202073)
Supplement: Supplemental data [file jci-136-202073-s282.pdf]

Supplemental material

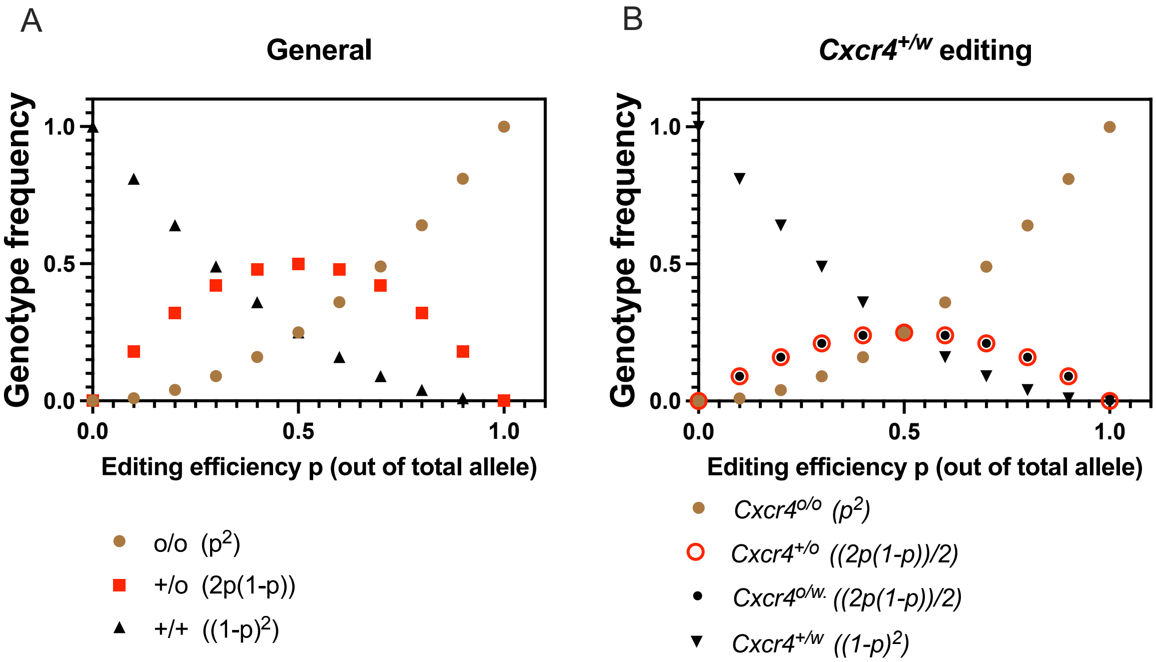

Supplementary Figure 1:

To estimate the frequencies of edited genotypes from the observed edited allele frequencies following gene editing, we applied the Hardy-Weinberg principle, assuming that editing occurs independently at each allele. **(A)** General case. Genotypes and the formula for calculating genotype frequencies from the Hardy-Weinberg equation ($p^2 + 2pq + q^2 = 1$) are coded at the bottom. p, frequency of the edited allele; q, frequency of the unedited allele (i.e., $q = 1 - p$).

(B) Specific case of *Cxcr4*$^{+/w}$ cells. The heterozygous population is composed of two genotypes: *Cxcr4*$^{o/w}$ (WT allele edited) and *Cxcr4*$^{+/o}$ (WHIM allele edited). Since both are assumed to occur with equal probability, each account for half of the heterozygous frequency, i.e., $2p(1 - p)/2$.
